# Twin peaks: The Omicron SARS-CoV-2 BA.1 and BA.2 epidemics in England

**DOI:** 10.1126/science.abq4411

**Published:** 2022-05-24

**Authors:** Paul Elliott, Oliver Eales, Nicholas Steyn, David Tang, Barbara Bodinier, Haowei Wang, Joshua Elliott, Matthew Whitaker, Christina Atchison, Peter J. Diggle, Andrew J. Page, Alexander J. Trotter, Deborah Ashby, Wendy Barclay, Graham Taylor, Helen Ward, Ara Darzi, Graham S. Cooke, Christl A. Donnelly, Marc Chadeau-Hyam

**Affiliations:** ^1^ School of Public Health, Imperial College London, London, UK.; ^2^ MRC Centre for Environment and Health, School of Public Health, Imperial College London, London, UK.; ^3^ Imperial College Healthcare NHS Trust, London, UK.; ^4^ National Institute for Health Research Imperial Biomedical Research Centre, London, UK.; ^5^ Health Data Research (HDR) UK, Imperial College London, London, UK.; ^6^ UK Dementia Research Institute, Imperial College London, London, UK.; ^7^ MRC Centre for Global infectious Disease Analysis and Jameel Institute, Imperial College London, London, UK.; ^8^ Department of Statistics, University of Oxford, Oxford, UK.; ^9^ Department of Mathematics, Imperial College London, London, UK.; ^10^ Department of Infectious Disease, Imperial College London, London, UK.; ^11^ CHICAS, Lancaster Medical School, Lancaster University, UK and Health Data Research, Lancaster, UK.; ^12^ Quadram Institute, Norwich, UK.; ^13^ Institute of Global Health Innovation, Imperial College London, London, UK.

## Abstract

Rapid transmission of severe acute respiratory syndrome coronavirus 2 (SARS-CoV-2) Omicron variant has led to record-breaking incidence rates around the world. The REal-time Assessment of Community Transmission-1 (REACT-1) study has tracked SARS-CoV-2 infection in England using reverse transcription polymerase chain reaction (RT-PCR) results from self-administered throat and nose swabs from randomly selected participants aged 5+ years, approximately monthly from May 2020 to March 2022. Weighted prevalence in March 2022 was the highest recorded in REACT-1 at 6.37% (N=109,181) with Omicron BA.2 largely replacing BA.1. Prevalence was increasing overall with the greatest increase in those aged 65-74 and 75+ years. This was associated with increased hospitalizations and deaths but at much lower levels than in previous waves against a backdrop of high levels of vaccination.

Since the emergence of Omicron as the dominant SARS-CoV-2 variant in England in mid- to late December 2021 (*1*, *2*), the peak in January 2022 associated with the BA.1 variant was the highest prevalence recorded in England to that time (*3*). This was followed by replacement of BA.1 by the more transmissible BA.2 (*3*–*5*). By late March 2022, BA.2 infections had surged in many European countries (*6*) and had become the predominant variant in the USA (*7*). Although Omicron infections led to fewer severe outcomes than Delta infections, the risk reduction depends on age (*8*) and background immunity, with some deaths observed in children under 12 years of age. In less vaccinated populations such as Hong Kong and Shanghai, with limited protection from vaccination among their oldest citizens (*9*), BA.2 has spread very quickly (*10*, *11*); the Omicron wave has caused over 97% of the total COVID-19 death toll in Hong Kong to date (with 9,146 deaths between 31 Dec 2021 and 13 May 2022 out of a total of 9,359 total between 31 Dec 2019 and 13 May 2022) (*12*, *13*).

In England, during the first phase of the Omicron (BA.1) epidemic (*1*), the country saw peaks in hospital admissions in late December 2021 to early January 2022 and in deaths (within 28 days of a positive test) in mid-January 2022 (*14*). Since then, following falls in February 2022, in late March 2022 hospital admissions returned to levels similar to those seen in January 2022 (~2000 admissions per day on average) with deaths also increasing in England since early March 2022.

The REal-time Assessment of Community Transmission-1 (REACT-1) study has tracked the spread of the SARS-CoV-2 virus among randomly selected community samples in England since May 2020 (*15*). Unlike reliance on testing of symptomatic individuals to estimate prevalence as is the case in most countries, the use of random samples of the population means that estimates are unbiased with respect to test-seeking behaviors, availability of tests and includes asymptomatic as well as symptomatic infections (*16*). With completion of the nineteenth and final round of REACT-1 data collection, we document here the transmission dynamics of SARS-CoV-2 in England since May 2020, in particular the emergence of the Omicron epidemic and the replacement of BA.1 and sub-lineages by BA.2.

## Overall prevalence and temporal trends

Of 697,055 individuals invited into the final (nineteenth) round of REACT-1, 109,181 (15.7%) registered and returned a throat and nasal swab (from March 8 to 31, 2022) with a valid SARS-CoV-2 reverse transcription polymerase chain reaction (RT-PCR) test result. Of these, 6,902 were positive, yielding a weighted prevalence of 6.37% [95% credible interval (CrI), 6.21%, 6.53%], the highest weighted prevalence observed throughout the REACT-1 study (Table 1).

**Table 1.** Unweighted and weighted prevalence of SARS-CoV-2 swab-positivity from REACT-1 across rounds 1 to 19.

**Round**	**Tested swabs**	**Positive swabs**	**Unweighted prevalence (95% CI)**	**Weighted prevalence (95% CrI)**	**First sample**	**Last sample**
1	120,620	159	0.13% (0.11%, 0.15%)	0.16% (0.13%, 0.19%)	01/05/20	01/06/20
2	159,199	123	0.08% (0.07%, 0.09%)	0.09% (0.07%, 0.11%)	19/06/20	07/07/20
3	162,821	54	0.03% (0.03%, 0.04%)	0.04% (0.03%, 0.05%)	24/07/20	11/08/20
4	154,325	137	0.09% (0.08%, 0.11%)	0.13% (0.01%, 0.15%)	20/08/20	08/09/20
5	174,949	824	0.47% (0.44%, 0.50%)	0.60% (0.55%, 0.71%)	18/09/20	05/10/20
6	160,175	1,732	1.08% (1.03%, 1.13%)	1.30% (1.21%, 1.39%)	16/10/20	02/11/20
7	168,181	1,299	0.77% (0.73%, 0.82%)	0.94% (0.87%, 1.01%)	13/11/20	03/12/20
8	167,642	2,282	1.36% (1.31%, 1.42%)	1.57% (1.49%, 1.66%)	06/01/21	22/01/21
9	165,456	689	0.42% (0.39%, 0.45%)	0.49% (0.44%, 0.55%)	04/02/21	23/02/21
10	140,844	227	0.16% (0.14%, 0.18%)	0.20% (0.17%, 0.23%)	11/03/21	30/03/21
11	127,408	115	0.09% (0.07%, 0.11%)	0.10% (0.08%, 0.13%)	15/04/21	03/05/21
12*	108,911	135	0.12% (0.10%, 0.15%)	0.15% (0.12%, 0.18%)	20/05/21	07/06/21
13	98,233	527	0.54% (0.49%, 0.58%)	0.63% (0.57%, 0.69%)	24/06/21	12/07/21
14**	100,527	764	0.76% (0.71%, 0.82%)	0.83% (0.76%, 0.89%)	09/09/21	27/09/21
15***	100,112	1,399	1.40% (1.33%, 1.47%)	1.57% (1.48%, 1.66%)	19/10/21	05/11/21
16****	97,089	1,192	1.23% (1.16%, 1.30%)	1.41% (1.33%, 1.51%)	23/11/21	14/12/21
17†	102,174	4,073	3.99% (3.87%, 4.11%)	4.41% (4.25%, 4.56%)	05/01/22	20/01/22
18‡§	94,950	2,731	2.88% (2.77%, 2.98%)	2.88% (2.76%, 3.00%)	08/02/22	01/03/22
19§	109,181	6,902	6.32% (6.18%, 6.47%)	6.37% (6.21%, 6.53%)	08/03/22	31/03/22

*Sampling strategy changed for round 12 and subsequent rounds. Therefore unweighted prevalence is not directly comparable with previous rounds

**Including N=509 samples from 28-30 September 2021. Sample handling changed in round 14. Therefore prevalence is not directly comparable with previous rounds

***Including N=93 samples (all negatives) from 6-8 November 2021, and N=86 samples with no collection/arrival dates

****Including N=661 samples (including 12 positives) from 15-17 December 2021. Swab positivity was assessed using a multiplex assay from round 16 onwards. Test diagnostic characteristics may slightly differ with previous rounds

†Including N=862 (including 36 positives) from 21-24 January 2022

‡Including N=685 (including 18 positives) from 2-4 March 2022

§Incentives were used in rounds 18 and 19 to increase the response rates in previously under-represented groups

Prevalence levels by demographic and other characteristics are shown in table S1A and S1B. Weighted prevalence during round 19 was higher: in households with one or more children at 7.55% (95% CrI, 7.22%, 7.89%) compared with those without children at 5.89% (95% CrI, 5.71%, 6.08%); among those reporting contact with a confirmed COVID-19 case at 17.8% (95% CrI, 17.2%, 18.5%) compared with those without such contact at 4.00% (95% CrI, 3.86%, 4.16%); and among those reporting ‘classic’ COVID-19 symptoms (loss or change of sense of smell or taste, fever, new persistent cough) at 27.6% (95% CrI, 26.7%, 28.5%) compared with those without symptoms at 2.60% (95% CrI, 2.47%, 2.74%). Our results did not show substantial differences in weighted prevalence in relation to smoking or vaping status.

Multivariable logistic regression models showed increased risk of swab-positivity in those living i) in larger households including 3 to 5 persons (vs. single-person households) with mutually adjusted Odds Ratio (OR) of 1.09 (95% CI 1.01, 1.17), and ii) with one or more children (vs. household without children) with mutually adjusted OR of 1.09 (1.01, 1.18) (table S2). Results also showed lower risk of swab-positivity in those living i) in urban (vs. rural) (*17*) areas with mutually adjusted OR of 0.93 (95% CI 0.88, 0.99) and ii) in deprived (Index of Multiple Deprivation (*18*) in the first and second quintiles) vs. most affluent (fifth quintile) areas with mutually adjusted OR of 0.82 (95% CI, 0.75, 0.90) and 0.91 (0.84, 0.98), respectively. This last finding is in contrast to our results in earlier rounds showing higher infection prevalence in more deprived areas (*19*), but is consistent with a report from the Office for National Statistics Coronavirus Infection Survey covering the two weeks up to April 23, 2022 (when BA.2 dominated), which also found higher infection prevalence in less deprived areas (*20*).

**Fig. 1.**
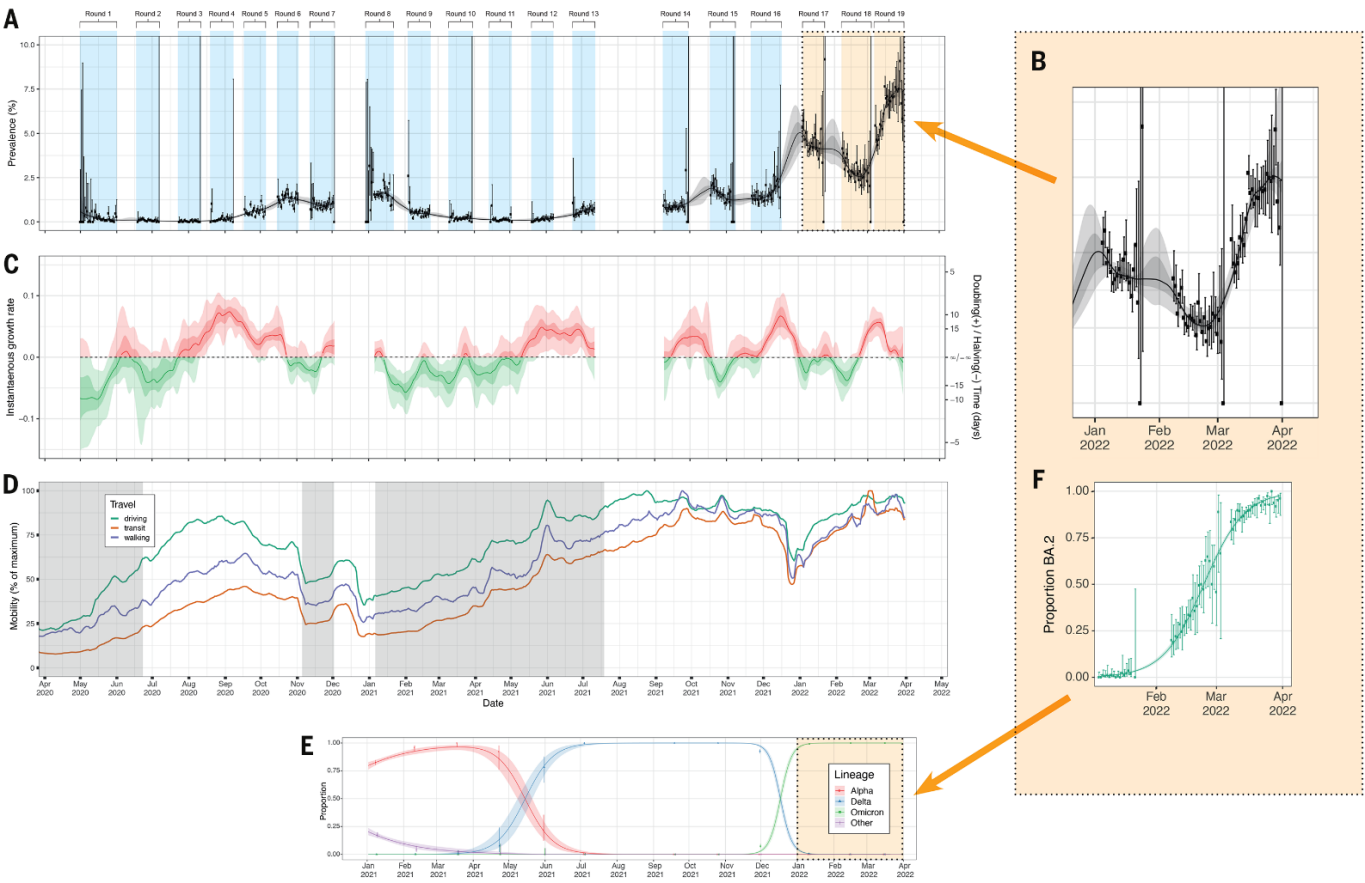
Overview of SARS-CoV-2 swab-positivity across the 19 rounds of REACT-1 study. (**A**) P-spline model fit to all rounds of REACT-1. Shaded grey region shows 50% (dark grey) and 95% (light grey) posterior credible interval for the P-spline model. Weighted prevalence of swab-positivity (Y axis) is represented for each day of sampling (X axis). Weighted observations (black dots) and 95% credible intervals (vertical lines) are also shown on an ordinal scale. (**B**) Blow up of the P-spline model for rounds 16 to 19. (**C**) Instantaneous growth rate for each of the swab days of the REACT study from the log-transformed P-spline. Posterior median estimates are represented as a smoothed solid line and 50% (dark shaded regions) and 95% (light shaded regions) are plotted in red for positive and in green for negative growth rates. (**D**) Daily Apple mobility indices for walking, driving and transit from phone location data for the duration of the REACT-1 study (1 May 2020 to 31 March 2022). We report seven-day moving averages of the indices and have scaled them to the maximum observed during the study period. Grey shaded regions represent periods when lockdown was implemented in England. (**E**) Daily proportion of Alpha (red), Delta (blue), Omicron (green) and other (purple) SARS-CoV-2 lineages across rounds 8 to 19 of the REACT-1 study. Mean daily proportions (solid lines) and their 95% credible intervals (shaded regions). (**F**) Daily proportion of BA.2 and its sub-lineages (vs all other Omicron sub-lineages) infections among positive swabs with determined lineage and at least 50% genome coverage in round 17, round 18 and round 19. Point estimates are represented (dots) along with 95% confidence intervals (vertical lines). Smoothed estimates of the proportion are also shown (solid line) together with their 95% credible intervals (shaded regions).

Trends in prevalence, growth rate, predominant variants, lockdown periods in England and mobility data over the 23 months from May 1, 2020 to March 31, 2022 are shown in Fig. 1, A to E. A P-spline model fit to all REACT-1 data shows an initial decline during the first lockdown in England, and increases in the second wave from autumn 2020 through January 2021, the Delta wave in summer to November 2021 and the initial Omicron wave from December 2021 to January 2022. We observed a fall in weighted prevalence (though still at high levels) during February 2022 followed by a steep increase in March 2022 (Fig. 1, A and B). We estimated doubling time in weighted prevalence of 30.5 (95% CrI 25.8, 37.0) days in round 19 (March 8 to 31, 2022), corresponding to a within-round *R* of 1.07 (95% CrI 1.06, 1.09) with >0.99 posterior probability that *R*>1 (Table 2).

**Table 2.** Growth rates per day (r), reproduction numbers (R) and doubling/halving times (in days) of SARS-CoV-2 swab-positivity from exponential model fits on data from round 19 (March 8 to 31, 2022).

		**Growth rate per day (r)**	Reproduction number (R)**	**Probability R>1, r>0**	**Doubling (+) / Halving (–) time (in days)**
All positives		0.023 (0.019, 0.027)	1.07 (1.06, 1.09)	>0.99	30.5 (37.0, 25.8)
Age	Aged 5 to 11	−0.017 (−0.029, −0.005)	0.94 (0.90, 0.98)	<0.01	−41.0 (−23.9, *)
	Aged 12 to 17	0.021 (0.003, 0.039)	1.07 (1.01, 1.13)	0.99	33.1 (*, 17.8)
	Aged 18 to 24	0.028 (0.013, 0.043)	1.09 (1.04, 1.14)	>0.99	24.6 (*, 16.2)
	Aged 25 to 34	0.028 (0.018, 0.038)	1.09 (1.06, 1.12)	>0.99	24.7 (38.7, 18.2)
	Aged 35 to 44	0.018 (0.007, 0.028)	1.06 (1.02, 1.09)	>0.99	39.4 (*, 24.8)
	Aged 45 to 54	0.008 (−0.002, 0.019)	1.03 (0.99, 1.06)	0.94	* (*, 37.2)
	Aged 55 to 64	0.029 (0.018, 0.041)	1.10 (1.06, 1.13)	>0.99	23.6 (38.8, 17.0)
	Aged 65 to 74	0.040 (0.027, 0.053)	1.13 (1.09, 1.17)	>0.99	17.3 (25.6, 13.1)
	Aged 75 and over	0.041 (0.024, 0.057)	1.13 (1.08, 1.19)	>0.99	17.1 (29.0, 12.2)
Region	East Midlands	0.019 (0.004, 0.033)	1.06 (1.01, 1.11)	0.99	37.1 (*, 21.0)
	West Midlands	0.022 (0.008, 0.036)	1.07 (1.03, 1.12)	>0.99	31.5 (*, 19.4)
	East of England	0.034 (0.022, 0.045)	1.11 (1.07, 1.15)	>0.99	20.7 (31.6, 15.5)
	London	0.004 (−0.007, 0.014)	1.01 (0.98, 1.05)	0.75	* (*, 49.7)
	North West	0.029 (0.017, 0.041)	1.09 (1.05, 1.13)	>0.99	24.2 (41.7, 17.1)
	North East	0.041 (0.020, 0.062)	1.14 (1.07, 1.20)	>0.99	16.8 (34.6, 11.2)
	South East	0.016 (0.006, 0.026)	1.05 (1.02, 1.08)	>0.99	42.9 (*, 27.0)
	South West	0.025 (0.013, 0.036)	1.08 (1.04, 1.12)	>0.99	28.3 (*, 19.4)
	Yorkshire and The Humber	0.045 (0.031, 0.059)	1.15 (1.10, 1.19)	>0.99	15.5 (22.1, 11.8)

*Doubling/halving time had an estimated magnitude greater than 50 days and so represented approximately constant prevalence

**Within-round T was calculated assuming an Omicron-specific Gamma-distributed generation time with mean 3.3 days and standard deviation of 3.5 days

From the estimated daily growth rates (Fig. 1C) we clearly show periods of rapid growth associated with i) the second wave in England as Alpha variant replaced wild-type in late summer and autumn 2020, ii) Delta replacing Alpha in late spring and summer 2021, iii) Omicron replacing Delta in November to December 2021, and iv) BA.2 replacing BA.1 during February and March 2022 (Fig. 1, E and F). The growth rate plateaued at 0.06 (95% CrI 0.03, 0.07) on ~March 10, 2022 (Fig. 1C). March 2022 also corresponded to a period of high and increasing mobility (Fig. 1D), with indices for driving, walking and transit by March 31 reaching, respectively, 92.9%, 85.0%, and 84.2% of the maximum observed throughout the study period.

**Fig. 2.**
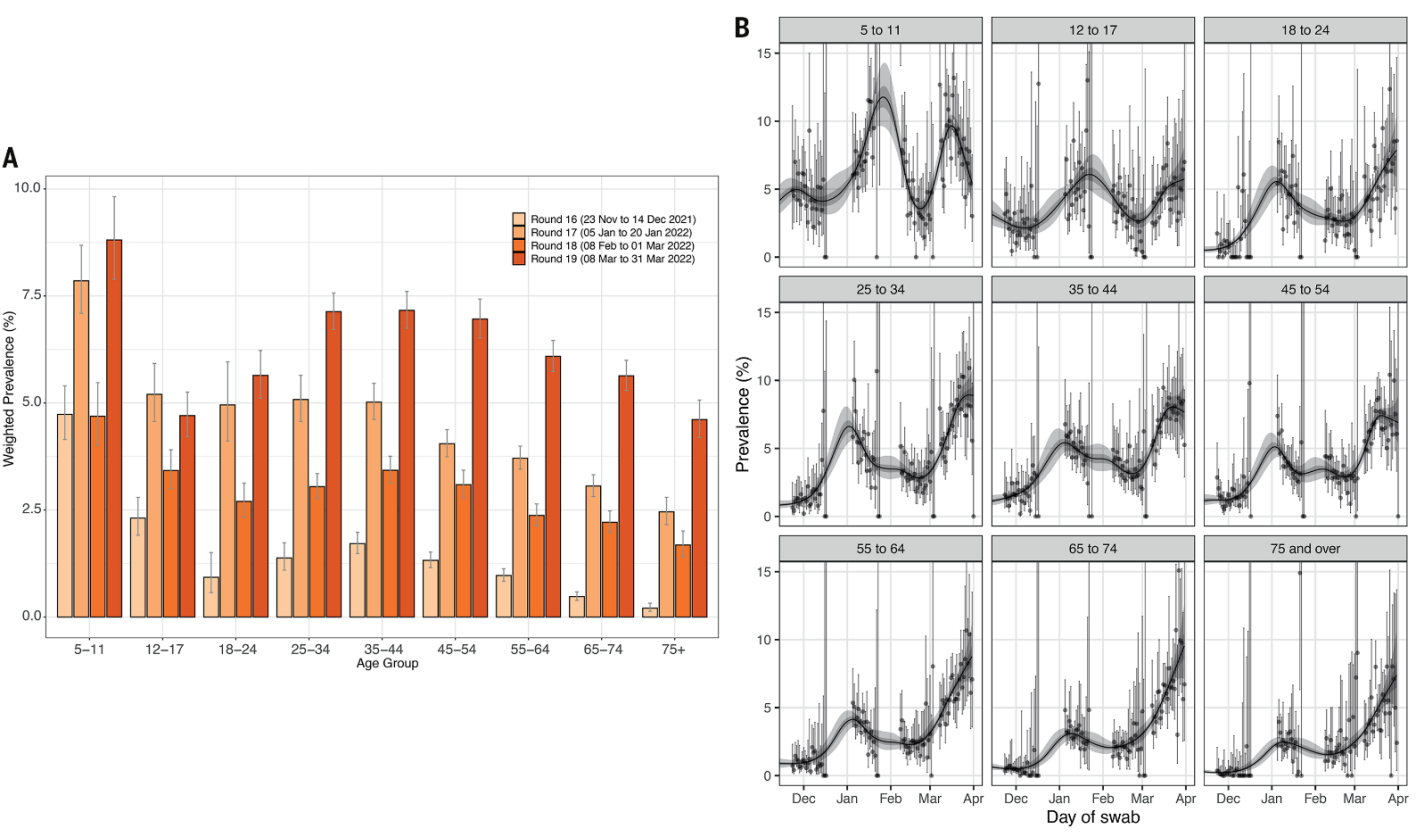
Weighted prevalence by age. (**A**) Weighted prevalence of SARS-CoV-2 swab-positivity by age group from round 16 (light orange) to round 19 (dark orange). Bars show the weighted prevalence point estimates and the vertical lines represent the 95% credible intervals. (**B**) Comparison of P-spline models fit to SARS-CoV-2 swab-positivity data from all rounds of REACT-1 for those ages 5-11, 12-17, 18-24, 25-34, 35-44, 45-54, 55,64, 65-74, and 75+ years. Shaded regions show 50% (dark shade) and 95% (light shade) posterior credible interval for the P-spline models. Results are presented for each day (X axis) of sampling for rounds 16, 17, 18, and 19, and the prevalence of swab-positivity is shown (Y axis). Weighted observations (dots) and 95% credible intervals (vertical lines) are also shown.

During the Omicron period, we observed peaks in weighted prevalence during round 17 (January 5 to 20, 2022) and round 19, with highest prevalence in both rounds at ages 5 to 11 years (Fig. 2A and table S1A). P-splines fit to daily weighted prevalence in nine age groups (Fig. 2B) indicated a steep increase in weighted prevalence between round 18 (February 8 to March 1, 2022) and round 19 at all ages, followed by i) a peak in those aged 5 to 11 years around March 16 or 17, 2022, with a subsequent fall; ii) a monotonic increase throughout round 19 in those aged 12 to 17, 18 to 24, 55 to 64, 65 to 74 and 75+ years; and iii) an indication that the weighted prevalence may have peaked by the end of March 2022, subsequently shown to be the case in the Office for National Statistics Coronavirus Infection Survey data (*21*). Exponential models fit to data from round 19 in the nine age groups showed an overall within-round decreasing prevalence in those aged 5 to 11 years with within-round R of 0.94 (95% CrI 0.90, 0.98) and <0.01 posterior probability that *R*>1, while there was a within-round increasing prevalence at all other ages (with ≥0.99 posterior probability that *R*>1) except for those aged 45 to 54 years (Table 2).

In round 13 (June 24 to July 12, 2021) (*22*) only 2.86% (weighted estimate) of children aged 12 to 17 years had been vaccinated. At that time, weighted prevalence of SARS-CoV-2 swab-positivity was 1.53 (95% CrI 1.00, 2.06) times higher among 12 to 17 year-olds than in those aged 5 to 11 years. Since then, as the vaccine program in older children in England took off, the ratio of weighted prevalence in children aged 12 to 17 years relative to that of 5 to 11 year-olds (almost all unvaccinated) dropped to 0.53 (95% CrI 0.28, 0.78) in round 19.

## Geographic trends

**Fig. 3.**
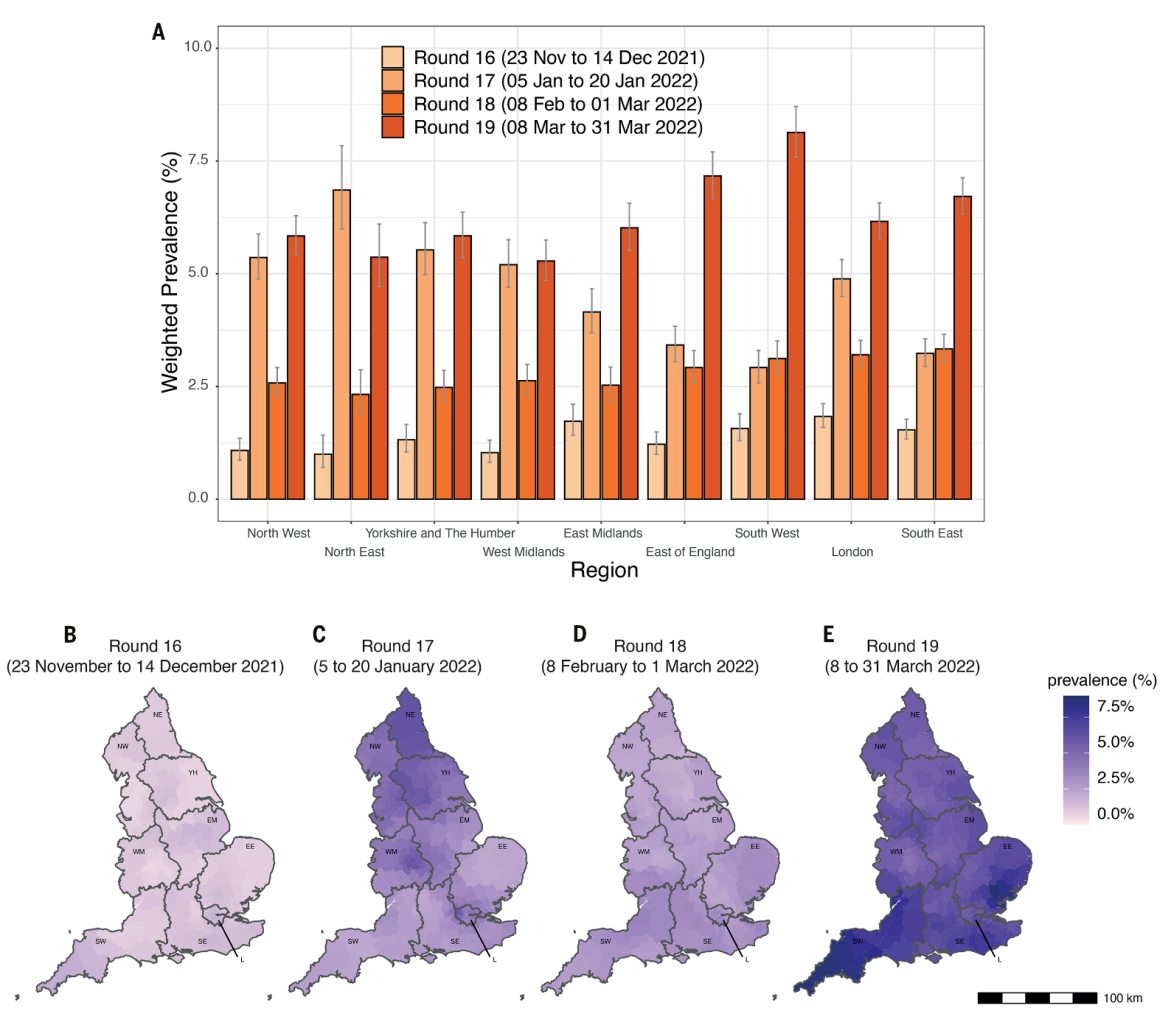
SARS-CoV-2 swab-positivity by region. (**A**) Weighted prevalence of SARS-CoV-2 swab-positivity by region from round 16 (light orange) to round 19 (dark orange). Bars show the weighted prevalence point estimates and the vertical lines represent the 95% credible intervals. (**B** to **E**) Neighborhood smoothed average SARS-CoV-2 swab-positivity prevalence by lower-tier local authority area for round 16 (B), round 17 (C), round 18 (D), and round 19 (E). Neighborhood prevalence calculated from nearest neighbors (the median number of neighbors within 30 km in the study). Average neighborhood prevalence displayed for individual lower-tier local authorities for the whole of England. Regions: NE = North East, NW = North West, YH = Yorkshire and The Humber, EM = East Midlands, WM = West Midlands, EE = East of England, L = London, SE = South East, SW = South West.

Region-specific weighted prevalence in round 19 ranged from 5.28% (95% CrI 4.85%, 5.75%) in West Midlands to 8.13% (95% CrI 7.59%, 8.71%) in South West (Fig. 3A and table S1A) with within-round 19 *R*>1 in all regions except London (Table 2). Nearest neighbor smoothed estimates (see Materials and methods) indicated ‘twin peaks’ in weighted prevalence in rounds 17 and 19. There was a strong North-to-South decreasing prevalence gradient in round 17 (Fig. 3C) but a strong South-to-North decreasing gradient in round 19 (Fig. 3E) with estimated smoothed prevalence >8.0% for 19 Lower-Tier Local Authorities (LTLAs), all in South West and East of England, consistent with our findings of higher rates in rural compared with urban areas.

## Viral genome sequencing

Viral genome sequencing of the 5,621 positive samples obtained in round 19 resulted in 4,445 (79.1%) determined lineages with more than 50% genome coverage; one (0.02%, 95% CI 0.00%, 0.13%) was AY.4 Delta sub-lineage while all others were Omicron sub-lineages (table S3). Among these 9.11% (95% CI 8.28%, 10.0%; N=405) corresponded to BA.1 or its sub-lineages, 90.6% (95% CI 89.7%, 91.4%; N=4,026) to BA.2 or its sub-lineages, and 0.29% (0.16%, 0.50%, N=13) to BA.3. Ten samples with >90% genome coverage were identified as BA.1/BA.2 recombinants (N=7 XE, N=2 XL, and N=1 XJ).

Using exponential models we estimated a daily growth rate advantage of 0.10 (0.10, 0.11) in the odds of BA.2 (vs all other Omicron sub-lineages), a 97.7% (95% CI 97.4%, 98.0%) proportion of BA.2 as of March 31, 2022 (Fig. 1F), and an estimated 56.4 (95% CrI 54.2, 58.7) days for the proportion of BA.2 to grow from 5% to 95%. This is an approximately two-fold lower rate than our estimate for the Delta-to-Omicron transition (28.5, 95% CI 26.3, 30.7 days), over 20% higher than the Alpha-to-Delta transition and almost four-fold higher than the wild-type-to-Alpha transition (Table 3).

**Table 3.** Growth rate advantage estimates from exponential growth models of the odds of the main SARS-CoV-2 (sub-) lineages. Point estimates and 95% credible intervals are reported along with the estimated time (in days) for the proportion of the lineage of interest to grow from 5 to 50% and from 5 to 95%. Results are presented for the Wild-type-to-Alpha, the Alpha-to-Delta, the Delta-to-Omicron, and the other Omicron-to-BA.2 sub-lineages transitions separately.

**Lineages competing**	**Growth rate advantage**	**Time (5% to 50%)**	**Time (5% to 95%)**
Alpha vs Wild-Type (Nov 2020-Apr 2021)	0.029 (0.019, 0.042)	100.1 (157.8, 70.5)	200.2 (315.5, 141.0)
Delta vs Alpha (Apr - Jul 2021)	0.085 (0.070, 0.104)	34.8 (42.2, 28.4)	69.6 (84.4, 56.8)
Omicron vs Delta (Dec 2021 -Jan 2022)	0.207 (0.192, 0.224)	14.2 (15.4, 13.1)	28.5 (30.7, 26.3)
BA.2 vs non-BA.2 Omicron (Jan - Apr 2022)	0.104 (0.100, 0.109)	28.2 (29.4, 27.1)	56.4 (58.7, 54.2)

## Discussion

Within a purpose-designed series of large cross-sectional population-based surveys with random selection of participants, we document here the transmission dynamics of SARS-CoV-2 in England from May 1, 2020 to March 31, 2022, at the height of the Omicron BA.2 wave. The Omicron epidemic in England was characterized by two distinct phases: i) very rapid replacement of Delta by Omicron during December 2021 and early January 2022, leading to the highest rates of infection since the start of REACT-1; ii) rapid replacement of Omicron BA.1 and sub-lineages by BA.2 during February to March 2022. Mobility indices also reached their highest levels (since October 2021) in March 2022, reflecting increased social mixing as restrictions were eased. Weighted prevalence in March 2022 increased most rapidly among older adults who – despite high levels of vaccination – remain the most vulnerable to serious illness, hospitalizations and death from COVID-19. Hospitalizations and deaths from COVID-19 in England also increased in March 2022 as infections were rising (*14*), but at much lower rates than in previous waves, reflecting the high levels of vaccination in the population (*14*).

We show a transmission advantage for Alpha compared to wild-type in the second wave of infections in England, peaking in January 2021, for Delta as it replaced Alpha during April-June 2021, for Omicron (BA.1) as it rapidly replaced Delta and most recently for BA.2 versus BA.1 and its sub-lineages. Data showing a transmission advantage for BA.2 compared to BA.1 have also been reported from the national routine testing data in the UK (*23*) and in Denmark (*24*).

The Omicron epidemic in England – involving ‘twin peaks’ as the epidemic transitioned from Delta to Omicron (BA.1) and then from BA.1 to BA.2 – has unfolded over a three-month period, ahead of similar epidemics in most other countries. While the immune landscape due both to natural infection and vaccination differs by country and over time (*16*), the transmission dynamics in England are highly relevant to other high-income countries that, like England, experienced Alpha, Delta and subsequently Omicron waves of infection alongside an extensive vaccination program.

The transmission advantage for one variant over another will depend on the immune background (which varies over time due to natural infection and vaccination) as well as transmissibility (*25*) and mean generation time so it is not possible to assess directly how much more intrinsically transmissible Omicron is compared to wild-type or Alpha; nonetheless, our data show that each new variant of concern has demonstrated a transmission advantage over the previous variants. In addition, we detected recombinant infections, notably XE (BA.1/BA.2). Little is known about the clinical manifestations of XE and whether it may lead to more severe disease than BA.1 or BA.2, but early indications suggest a growth advantage compared with BA.2 (*26*). Continued surveillance of such recombinant infections is warranted.

To deal with the initial phase of the Omicron epidemic, some countries re-initiated social distancing policies (*27*), while in the USA healthcare systems struggled to cope with the increase in healthcare demands (*28*) and in England the vaccination program was accelerated. Subsequently, the UK government (on February 24, 2022) removed all domestic legal restrictions concerning COVID-19 in England (*29*) as part of the government’s plan for ‘Living with COVID-19’; (*30*) from February 24, 2022 legal requirement to self-isolate for COVID-19 was lifted and since April 1, 2022, all remaining restrictions in England were removed (*29*). Also, from April 1, 2022, with a few exceptions, free lateral flow and PCR tests were no longer available, and other surveillance measures were curtailed, with greater reliance on the vaccine program to manage the ongoing epidemic. In this regard, our most recent data on infections in children show, through much of March 2022, much higher infection rates in 5-to-11 year olds (for whom vaccination rollout only commenced April 2022) than 12-to-17 year olds, over 70% of whom had been vaccinated (one or two doses) by end of March 2022 (*31*). The drop in the prevalence in swab-positivity among the 5-to-11 year olds from mid-March 2022 from very high levels over a prolonged period, could suggest some depletion, at least temporarily, in their population-level susceptibility due to high levels of natural infection.

Our study has limitations. We rely on unsupervised, self-swabbing at home by named individuals selected at random from the National Health Service (NHS) registers. While response rates of over 30% were achieved during the first lockdown in England in May 2020, they fell to 12.2% by round 17 (January 2022). We included a small monetary incentive in rounds 18 and 19 (February to March 2022) among participants aged 13 to 44 years, which increased overall response rates in those rounds to ~15.0%. Additionally, we used within-round random iterative method (rim) weighting (*32*) to correct the sample to be representative of the base population. During the 23 months of the study, we have adapted the way samples were handled (for example, courier to post, no cold chain, inclusion of a multiplex PCR assay in the latter rounds). Although these changes may have introduced small effects into between-round comparisons, they should not have affected within-round trends.

In conclusion, we report unprecedented and increasing prevalence of SARS-CoV-2 infections in England during March 2022. We observed Omicron ‘twin peaks’ as BA.1 replaced Delta and BA.2 replaced BA.1, while at the same time, society opened up with all legal restrictions related to COVID-19 in England lifted as part of its ‘Living with COVID-19’ strategy. These high rates of infections were associated with increasing hospitalizations and deaths due to COVID-19 in England during March 2022, but at much lower levels than in previous waves against a backdrop of high levels of vaccination in the population. These transmission dynamics in England may be relevant to the experience in the USA and other countries as BA.2 takes hold as the predominant variant worldwide.

## Materials and methods

### Study design

The REACT-1 study involved a series of cross-sectional surveys of random samples of the population of England at ages 5+ years (*15*), carried out over 19 distinct rounds from May 1, 2020 to March 31, 2022. Those registering for the study were sent a self-administered throat and nose swab kit with instructions and asked to complete a questionnaire. In total, 2,512,797 participants had a valid test result for SARS-CoV-2 by RT-PCR across the 19 rounds of the study (Table 1) from among 14,036,117 individuals who were sent invitation letters, giving an overall response rate of 17.9% (completed tests/letters sent out). We focus here on the Omicron period spanning round 16 (November 23 to December 14, 2021, N=97,089), round 17 (January 5 to 20, 2022, N=102,174), round 18 (February 8 to March 1, 2022, N=94,950) and round 19 (March 8-31, 2022, N=109,181).

The sampling frame was the NHS general practitioner list of patients in England (covering almost the entire population) which includes name, address, age and sex. Participants provided information on ethnicity, household size, occupation, symptoms and other variables (*33*). We used residential postcode to link to an area-level Index of Multiple Deprivation (an overall relative measure of deprivation) (*18*) and to urban/rural status (*17*). We added small incentives in rounds 18 and 19 to increase response rates among under-represented groups. We used a multiplex including influenza A and B for rounds 16 to 19; only the SARS-CoV-2 results are reported here.

Initially we aimed to obtain approximately equal numbers of participants in each LTLA in England (N=315), but from round 12 (May 20 to June 7, 2021) we switched to obtaining a random sample in proportion to population size at LTLA level. We use rim weighting (*32*) to provide prevalence estimates for the population of England as a whole, adjusting for age, sex, deciles of the Index of Multiple Deprivation, LTLA counts, and ethnic group. Incentives were added to improve response among under-represented groups in rounds 18 and 19. For return of a completed test, a gift voucher worth £10 was offered to those aged 13 to 17 and 35 to 44 years and £20 to those aged 18 to 34 years.

Up to round 13 (June 24 to July 12, 2021), we collected dry swabs sent by courier to the laboratory on a cold chain but from round 14 (September 9 to 27, 2021 including 509 samples from 28-30 September) we switched to ‘wet’ (saline) swabs which (round 14) were sent to the laboratory either by courier (no cold chain) or priority post, and from round 15 (October 19 to November 5, 2021) onwards by priority post only. Because of delays in the post for return of swabs, we include a small proportion of samples obtained after the nominated closing date for each of rounds 14 to 18.

A test result was positive if both N gene and E gene targets were detected or N gene was detected with cycle threshold (Ct) value below 37.

### Viral genome sequencing

We carried out viral genome sequencing (Quadram Institute, Norwich, UK) of positive samples with Ct ≤34 for either E or N gene. We used the ARTIC protocol (*34*) (version 4 for rounds 16 and 17 and version 4.1 for rounds 18 and 19) for viral RNA amplification, CoronaHiT for preparation of sequencing libraries (*35*), the ARTIC bioinformatics pipeline (*34*) and assigned lineages using Pangolin (v4.0 with pangolin-data v1.2.133) (*36*).

RT-PCR was performed on 96 randomly chosen samples using the CDC assay (*37*) by the Quadram Institute as a secondary confirmation of the Ct values.

### Data analyses

As noted, we used rim weighting (*32*) to estimate round-specific weighted prevalence and 95% credible intervals. We used logistic regression to estimate the odds of testing positive by employment, ethnicity, household size, children in household, smoking and vaping status, urban/rural status, and deprivation, adjusting for age, region and subsequently, all other variables examined. We fit a Bayesian penalized-spline (P-spline) model (*38*, *39*) to the daily data to visualize temporal trends in swab-positivity over the whole study period. Additionally, we fitted nine age-group-specific P-splines (5 to 11, 12-17, 18 to 24, 25 to 34, 35 to 44, 45 to 54, 55 to 64, 65 to 74 and 75+ years) with the smoothing parameter obtained from the model fit to all the data. To fit the P-splines we used a No-U-Turn Sampler in logit space (*40*), partitioning the data into approximately 5-day sections by regularly spaced knots, and minimizing edge effects by adding further knots beyond the study period. Models were implemented and run using Rstan (*41*). We used day of swabbing where reported or otherwise day of pick-up by courier or first Post Office scan where available. We guarded against over-fitting by use of fourth-order basis splines (b-splines) over the knots including a second-order random-walk prior distribution on the coefficients of the b-splines; the prior distribution penalized against changes in growth rate unless supported by the data (*39*).

We estimated r, the daily overall exponential growth/decay rate (for SARS-CoV-2 swab-positivity), over the entire period of the study (since May 1, 2020) (*39*). The reproduction number, *R*, for the Omicron period was estimated assuming a gamma-distributed generation time with Omicron-specific mean 3.3 days and standard deviation 3.5 days (shape n=0.89 and rate β=0.27) as (*42*):


$$R={\left(1+\frac{r}{\beta }\right)}^{n}$$

The use of prevalent swab-positivity data means that changes in the underlying exponential growth rate of new infections are not detected immediately. Instead, following a change in the underlying growth rate, the estimates move smoothly between the previous value and the more recent one, regardless of the underlying mixture of variants and sub-lineages.

We estimated the growth rate advantage (a comparison of variant-specific growth rates) for the transition from wild-type to Alpha, Alpha to Delta, Delta to Omicron and Omicron BA.1 (and sub-lineages) to BA.2 by fitting a Bayesian logistic regression model to the daily proportions of the competing variants. The daily relative growth rates in the log-odds of Alpha, Delta, Omicron and other lineages were estimated, assuming constant growth rates, using a Bayesian multinomial logistic regression model fit to the categorical outcome variable (Alpha, Delta, Omicron, other) over rounds 8 to 19 with Delta set as the reference category.

The time taken for the proportion of one lineage to increase from 5% to 50% was calculated assuming only two lineages were present and using the pairwise difference in their growth rates, r*, in the equation:


$$T_{5\%\;\text{to }50\%}=\frac{\text{log}\left(\frac{0.05}{1-0.05}\right)-\text{log}\left(\frac{0.5}{1-0.5}\right)}{r*}$$

For example, in calculating Delta’s rise against Alpha, r* would be the difference in growth rates of Alpha and Delta. Due to the assumed symmetry, the time of one lineage to increase from 5% to 95% is two times *T*_5% to 50%_.

In order to account for participants’ proximity across LTLA and heterogeneous population density across LTLAs, we calculated smoothed prevalence per LTLA using a nearest neighbor approach. Briefly, for each LTLA, we estimated the median number of participants (M) within 30 km of each other. Then for a random sample of 15 participants per LTLA, we calculated the prevalence of infection among the nearest M people. The smoothed prevalence by LTLA is then defined as the average of the 15 prevalence estimates in that area.

Statistical analyses were performed with R software, version 4.0.5.

### Mobility data

Daily data on mobility (transit, driving and walking) were downloaded for England from Apple Mobility Trends Report (*43*). Seven-day moving averages, relative to the maximum seven-day average between May 1, 2020 and March 31, 2022, were plotted on the fourth of the seven days. Days were defined from midnight to midnight, US Pacific time. Random rotating identifiers, rather than Apple IDs, are used for data sent from Apple users to the Apple Maps service. Thus no profiles are collected on individual movements. As Apple Maps thus has no demographic data on users, it is not possible to assess the representativeness of the mobility data provided.

### Lockdown dates and restrictions in England

In Fig. 1D grey shaded regions represent periods when lockdown was implemented in England. The following dates were used as start and end dates (from May 1, 2020 to March 31, 2022):

June 23, 2020: The first national lockdown was announced on March 23, 2020 (*44*). The Prime Minister announced key changes to lockdown restrictions on June 23, 2020 (*45*).

November 5, 2020: The second national lockdown in England was announced on November 5, 2020 (*46*).

December 2, 2020: The second national lockdown in England ended on December 2, 2020 (*47*) after four weeks. England moved to a stricter three-tiered system of restrictions.

January 6, 2021: The third national lockdown in England was announced on January 6, 2021 (*48*).

July 18, 2021: On March 8, 2021 England started a phased release of lockdown regulations (*49*). Lockdown laws ceased to be in force on July 18, 2021 (*50*).

February 24, 2022: All domestic legal restrictions concerning COVID-19 in England were removed (*29*).

April 1, 2022: All remaining restrictions in England were removed (*29*).

The easing of restrictions during or after lockdown was not complete at a single time point so these dates up to April 1, 2022 should not be regarded as representing presence or absence of all restrictions.

## Supplementary Materials

### Other Supplementary Material for this manuscript includes the following:

MDAR Reproducibility Checklist
