# Supplementary material for: Twin peaks: The Omicron SARS-CoV-2 BA.1 and BA.2 epidemics in England
Source: Science. 2022 May 24:eabq4411. doi: 10.1126/science.abq4411 (PMC9161371; doi:10.1126/science.abq4411)
Supplement: Supplementary file 1 — Supplementary Text Tables S1 to S3 [file science.abq4411_sm.pdf]

Science
AAAS

Supplementary Materials for

Twin peaks: The Omicron SARS-CoV-2 BA.1 and BA.2 epidemics in England

Paul Elliott *et al.*

Corresponding authors: Paul Elliott, p.elliott@imperial.ac.uk; Christl A. Donnelly, c.donnelly@imperial.ac.uk; Marc Chadeau-Hyam, m.chadeau@imperial.ac.uk

DOI: 10.1126/science.abq4411

The PDF file includes:

Supplementary Text
Tables S1 to S3

Other Supplementary Material for this manuscript includes the following:

MDAR Reproducibility Checklist

Supplementary Text

Link to questionnaires

The full questionnaire for Round 19 is available here:

https://www.imperial.ac.uk/media/imperial-college/institute-of-global-health-innovation/REACT1_Round-19_ANTIGEN-Individual-Questionnaire_March-2022.pdf

Those for other rounds can be found here:

https://www.imperial.ac.uk/medicine/research-and-impact/groups/react-study/for-researchers/react-1-study-materials/

Table S1A. Weighted prevalence of SARS-CoV-2 swab-positivity in rounds 18 and 19 by sex, age, region, urban/rural area, employment, and ethnic group.

		Round 18			Round 19		
Variable		Positive	Total	Weighted Prevalence	Positive	Total	Weighted Prevalence
Sex	Male	1,199	40,936	2.97% (2.80%, 3.15%)	2,960	47,514	6.41% (6.17%, 6.66%)
	Female	1,532	54,005	2.79% (2.64%, 2.95%)	3,942	61,663	6.32% (6.12%, 6.54%)
	Unknown	0	9	*	0	4	*
Age	05-11	161	3,392	4.69% (4.01%, 5.48%)	319	3,627	8.81% (7.89%, 9.82%)
	12-17	261	7,584	3.42% (3.00%, 3.91%)	428	9,132	4.71% (4.21%, 5.25%)
	18-24	205	7,407	2.70% (2.33%, 3.13%)	444	7,705	5.65% (5.11%, 6.23%)
	25-34	463	14,970	3.05% (2.77%, 3.35%)	1,150	15,620	7.13% (6.72%, 7.57%)
	35-44	492	14,386	3.43% (3.13%, 3.76%)	1,115	15,206	7.16% (6.75%, 7.61%)
	45-54	371	11,976	3.09% (2.79%, 3.43%)	920	13,055	6.96% (6.52%, 7.43%)
	55-64	343	14,405	2.37% (2.13%, 2.64%)	1,094	17,897	6.09% (5.74%, 6.46%)
	65-74	308	13,635	2.21% (1.98%, 2.48%)	981	17,321	5.63% (5.29%, 6.00%)
	75+	127	7,195	1.68% (1.41%, 2.01%)	451	9,618	4.61% (4.20%, 5.07%)
Region	North West	291	11,386	2.58% (2.28%, 2.92%)	787	13,281	5.84% (5.42%, 6.29%)
	North East	100	4,364	2.33% (1.88%, 2.87%)	263	4,987	5.37% (4.72%, 6.10%)
	Yorkshire and The Humber	229	9,133	2.48% (2.15%, 2.85%)	615	10,678	5.84% (5.36%, 6.36%)
	West Midlands	264	9,745	2.63% (2.31%, 2.99%)	617	11,471	5.28% (4.85%, 5.75%)
	East Midlands	211	8,295	2.53% (2.18%, 2.93%)	582	9,763	6.02% (5.51%, 6.56%)
	East of England	298	10,658	2.92% (2.58%, 3.29%)	845	12,102	7.17% (6.67%, 7.70%)
	South West	316	9,911	3.12% (2.77%, 3.51%)	908	11,479	8.13% (7.59%, 8.71%)
	London	508	15,480	3.20% (2.92%, 3.52%)	1,065	17,061	6.16% (5.77%, 6.57%)
	South East	514	15,978	3.33% (3.04%, 3.66%)	1,220	18,359	6.72% (6.32%, 7.13%)
Living in urban area	Yes	2,217	76,453	2.92% (2.80%, 3.06%)	5,418	87,429	6.22% (6.05%, 6.40%)
	No	501	18,249	2.68% (2.44%, 2.94%)	1,473	21,487	6.97% (6.60%, 7.35%)
	Unknown	13	248	3.90% (2.22%, 6.75%)	11	265	4.63% (2.41%, 8.74%)
Employment type	Health care or care home worker	269	7,812	3.27% (2.87%, 3.73%)	623	8,659	7.27% (6.66%, 7.93%)
	Other essential/key worker	418	13,396	3.14% (2.83%, 3.48%)	1,052	14,134	7.29% (6.83%, 7.77%)
	Other worker	1,093	37,420	3.01% (2.83%, 3.21%)	2,804	41,832	6.70% (6.44%, 6.97%)
	Not full-time, part-time, or self-employed	885	34,073	2.54% (2.36%, 2.72%)	2,284	42,132	5.44% (5.20%, 5.68%)
	Unknown	66	2,249	2.79% (2.16%, 3.60%)	139	2,424	6.02% (5.06%, 7.16%)
Ethnic group	White	2,346	80,897	2.90% (2.78%, 3.03%)	6,014	93,599	6.49% (6.32%, 6.67%)
	Asian	194	6,642	2.95% (2.52%, 3.43%)	399	7,463	5.26% (4.71%, 5.87%)
	Black	44	2,063	2.27% (1.66%, 3.10%)	114	2,140	5.43% (4.44%, 6.63%)
	Mixed	63	1,997	3.29% (2.50%, 4.31%)	141	2,188	6.80% (5.65%, 8.18%)
	Other	29	1,083	2.94% (1.97%, 4.36%)	70	1,163	5.86% (4.53%, 7.54%)
	Unknown	55	2,268	2.27% (1.70%, 3.02%)	164	2,628	6.16% (5.24%, 7.24%)

* Prevalence estimates are not reported if based on less than 10 observations

Table S1B. Weighted prevalence of SARS-CoV-2 swab-positivity in round 18 and round 19 by household size, number of children in the household, smoking and vaping status, contact with a COVID-19 case, reported previous COVID-19, protective behaviours, symptom status.

		Round 18			Round 19		
Variable		Positive	Total	Weighted Prevalence	Positive	Total	Weighted Prevalence
Household size	1	288	14,239	1.87% (1.65%, 2.11%)	914	17,093	5.26% (4.92%, 5.63%)
	2	845	33,450	2.50% (2.33%, 2.68%)	2,422	39,996	6.03% (5.79%, 6.28%)
	3	543	18,194	3.06% (2.79%, 3.35%)	1,416	20,059	6.91% (6.54%, 7.30%)
	4	716	19,042	3.88% (3.58%, 4.20%)	1,475	21,416	7.13% (6.74%, 7.53%)
	5	219	6,570	3.33% (2.87%, 3.85%)	462	7,051	6.97% (6.28%, 7.73%)
	6+	120	3,455	3.36% (2.76%, 4.09%)	213	3,566	6.02% (5.17%, 6.99%)
Number of children in the household	0	1,407	58,734	2.31% (2.19%, 2.44%)	4,114	69,000	5.89% (5.71%, 6.08%)
	1+	1,072	28,474	3.88% (3.63%, 4.14%)	2,334	30,809	7.55% (7.22%, 7.89%)
	Unknown	252	7,742	3.16% (2.78%, 3.60%)	454	9,372	4.72% (4.29%, 5.19%)
Smoking status	Never	1,229	43,924	2.70% (2.55%, 2.87%)	3,364	51,182	6.49% (6.27%, 6.72%)
	Former	604	21,844	2.70% (2.49%, 2.93%)	1,720	26,598	6.36% (6.06%, 6.68%)
	Current	129	5,057	2.63% (2.19%, 3.14%)	341	5,744	5.70% (5.10%, 6.36%)
	Unknown	769	24,125	3.33% (3.08%, 3.61%)	1,477	25,657	6.30% (5.95%, 6.67%)
Vaping status	Never	1,760	63,668	2.68% (2.55%, 2.81%)	4,912	75,246	6.43% (6.25%, 6.62%)
	Former	103	3,670	2.87% (2.34%, 3.51%)	265	4,213	5.97% (5.27%, 6.76%)
	Current	109	4,079	2.70% (2.22%, 3.28%)	294	4,762	6.22% (5.52%, 7.01%)
	Unknown	759	23,533	3.37% (3.11%, 3.65%)	1,431	24,960	6.28% (5.93%, 6.66%)
COVID case contact	No	1,187	69,708	1.73% (1.62%, 1.84%)	3,130	78,334	4.00% (3.86%, 4.16%)
	Yes, contact with a confirmed/tested COVID-19 case	1,056	10,312	10.35% (9.72%, 11.02%)	2,724	15,387	17.84% (17.17%, 18.52%)
	Yes, contact with a suspected COVID-19 case	142	2,098	6.86% (5.77%, 8.15%)	398	3,243	12.50% (11.28%, 13.83%)
	Unknown	346	12,832	2.55% (2.27%, 2.85%)	650	12,217	5.29% (4.87%, 5.74%)
Shielding	Yes	426	17,277	2.39% (2.16%, 2.65%)	1,216	20,353	6.00% (5.66%, 6.37%)
	No	1,953	64,720	3.08% (2.93%, 3.23%)	5,021	76,481	6.64% (6.45%, 6.84%)
	Unknown	352	12,953	2.58% (2.31%, 2.89%)	665	12,347	5.33% (4.91%, 5.78%)
Frequency wearing mask indoors	Always	717	24,303	2.85% (2.64%, 3.08%)	1,196	17,895	6.59% (6.21%, 6.99%)
	Sometimes	1,111	37,147	3.01% (2.83%, 3.20%)	2,767	39,189	7.07% (6.80%, 7.35%)
	Hardly ever	109	3,901	2.89% (2.37%, 3.52%)	326	5,254	6.12% (5.45%, 6.87%)
	Never	36	1,378	2.40% (1.70%, 3.40%)	89	1,595	5.48% (4.40%, 6.80%)
	Unknown	758	28,221	2.77% (2.56%, 3.00%)	2,524	45,248	5.81% (5.56%, 6.06%)
Symptom status	Classic COVID symptoms*	1,175	7,845	15.06% (14.21%, 15.95%)	3,230	11,602	27.57% (26.68%, 28.47%)
	Other symptoms	530	13,651	3.85% (3.52%, 4.22%)	1,343	16,963	7.87% (7.43%, 8.33%)
	No symptoms	682	60,676	1.17% (1.08%, 1.27%)	1,687	68,450	2.60% (2.47%, 2.74%)
	Unknown	344	12,778	2.53% (2.26%, 2.84%)	642	12,166	5.21% (4.80%, 5.66%)

* Classic COVID symptoms: loss or change of sense of smell or taste, fever, new persistent cough

Table S2. Multivariable logistic regression for round 19. Results are presented as Odds Ratios (95% confidence interval) adjusted for age and sex and additionally for region and all other variables (mutually adjusted OR).

	Category	Round 19	
		Adjusted for age and sex	Mutually adjusted*
Employment type	Health care or care home worker	1.04 (0.95, 1.14)	1.05 (0.96, 1.15)
	Other essential/key worker	1.08 (1.00, 1.16)	1.07 (0.99, 1.15)
	Other worker	Ref	Ref
	Not full-time, part-time, or self-employed	0.92 (0.86, 0.98)	0.90 (0.84, 0.96)
	Unknown	0.97 (0.81, 1.16)	0.98 (0.82, 1.18)
Ethnic group	White	Ref	Ref
	Asian	0.78 (0.70, 0.86)	0.82 (0.73, 0.91)
	Black	0.78 (0.64, 0.94)	0.83 (0.69, 1.01)
	Mixed	0.94 (0.79, 1.12)	0.97 (0.81, 1.15)
	Other	0.88 (0.69, 1.12)	0.92 (0.72, 1.18)
	Unknown	0.99 (0.84, 1.16)	1.03 (0.88, 1.21)
Household size	1-2	Ref	Ref
	3-5	1.15 (1.08, 1.22)	1.09 (1.01, 1.17)
	6+	1.04 (0.90, 1.21)	1.07 (0.91, 1.25)
Number of children in the household	0	Ref	Ref
	1+	1.15 (1.08, 1.23)	1.09 (1.01, 1.18)
	Unknown	1.05 (0.88, 1.25)	1.07 (0.90, 1.28)
Smoking status	Never	Ref	Ref
	Former	1.02 (0.96, 1.08)	1.03 (0.96, 1.10)
	Current	0.86 (0.77, 0.97)	0.91 (0.80, 1.04)
	Unknown	0.88 (0.81, 0.95)	0.95 (0.76, 1.19)
Vaping status	Never	Ref	Ref
	Former	0.91 (0.80, 1.03)	0.95 (0.83, 1.09)
	Current	0.90 (0.79, 1.01)	0.92 (0.81, 1.05)
	Unknown	0.87 (0.80, 0.94)	0.90 (0.72, 1.14)
Living in urban area	Yes	0.86 (0.81, 0.92)	0.93 (0.88, 0.99)
	No	Ref	Ref
	Unknown	0.53 (0.29, 0.97)	0.58 (0.31, 1.06)
Deprivation	1 Most deprived	0.74 (0.68, 0.81)	0.82 (0.75, 0.90)
	2	0.86 (0.80, 0.93)	0.91 (0.84, 0.98)
	3	0.96 (0.89, 1.03)	0.97 (0.91, 1.04)
	4	0.94 (0.88, 1.01)	0.95 (0.88, 1.01)
	5 Least deprived	Ref	Ref

*Odds ratios are mutually adjusted for all variables shown and for age and region

Table S3. Proportion of each of the N=4,445 SARS-CoV-2 lineages detected in positive samples with at least 50% genome coverage from round 19. Results are based on 5,621 positive sequenced samples.

Lineage	N (4,445)	Percentage
Omicron sub-lineage	4,444	99.9% (99.9% , 100.0%)
BA.1	27	0.61% (0.42% , 0.88%)
BA.1.1	262	5.89% (5.24% , 6.63%)
BA.1.1.1	14	0.32% (0.19% , 0.53%)
BA.1.1.10	1	0.02% (0.00% , 0.13%)
BA.1.1.12	6	0.14% (0.06% , 0.29%)
BA.1.1.13	6	0.14% (0.06% , 0.29%)
BA.1.1.14	11	0.25% (0.14% , 0.44%)
BA.1.1.15	11	0.25% (0.14% , 0.44%)
BA.1.1.4	1	0.02% (0.00% , 0.13%)
BA.1.1.9	1	0.02% (0.00% , 0.13%)
BA.1.10	2	0.05% (0.01% , 0.16%)
BA.1.12	1	0.02% (0.00% , 0.13%)
BA.1.13	3	0.07% (0.02% , 0.20%)
BA.1.15	5	0.11% (0.05% , 0.26%)
BA.1.15.1	4	0.09% (0.04% , 0.23%)
BA.1.16	7	0.16% (0.08% , 0.32%)
BA.1.17	41	0.92% (0.68% , 1.25%)
BA.1.17.2	1	0.02% (0.00% , 0.13%)
BA.1.5	1	0.02% (0.00% , 0.13%)
BA.2	3,676	82.7% (81.56% , 83.78%)
BA.2.1	124	2.79% (2.34% , 3.32%)
BA.2.10	9	0.20% (0.11% , 0.38%)
BA.2.12	12	0.27% (0.15% , 0.47%)
BA.2.2	5	0.11% (0.05% , 0.26%)
BA.2.3	165	3.71% (3.20% , 4.31%)
BA.2.8	3	0.07% (0.02% , 0.20%)
BA.2.9	32	0.72% (0.51% , 1.01%)
BA.3	13	0.29% (0.17% , 0.50%)
Delta sub-lineage		
AY.4	1	0.02% (0.00% , 0.13%)

We also detected (N=10) BA.1/BA.2 recombinants: 7 XE, 2 XL, and 1 XJ.
